# A population-based cohort study of chest x-ray screening in smokers: lung cancer detection findings and follow-up

**DOI:** 10.1186/1471-2407-12-18

**Published:** 2012-01-17

**Authors:** Lorenzo Dominioni, Nicola Rotolo, William Mantovani, Albino Poli, Salvatore Pisani, Valentina Conti, Massimo Paolucci, Fausto Sessa, Antonio Paddeu, Vincenzo D'Ambrosio, Andrea Imperatori

**Affiliations:** 1 Center for Thoracic Surgery, University of Insubria, Via Guicciardini, 9, 21100 Varese, Italy; 2 Department of Public Health and Community Medicine, University of Verona, Strada Le Grazie 8, 37134 Verona, Italy; 3 Epidemiology Observatory, Varese Local Health Authority, Via O. Rossi 9, 21100 Varese, Italy; 4 Department of Radiology, Ospedale S. Antonio Abate, Via Pastori 4, 21013 Gallarate, Italy; 5 Department of Human Morphology, University of Insubria, Via Monte Generoso 71, 21100 Varese, Italy; 6 Respiratory Care Unit, Department of Medicine, Ospedale S. Anna, Via Ravona, 22020 San Fermo della Battaglia, Como, Italy; 7 Thoracic Medicine Unit, Department of Medicine, Ospedale S. Antonio Abate, Via Pastori 4, 21013 Gallarate, Italy

**Keywords:** Lung cancer, Chest x-ray screening, Population-based, Cohort study, volunteer effect, Survival, Community

## Abstract

**Background:**

Case-control studies of mass screening for lung cancer (LC) by chest x-rays (CXR) performed in the 1990s in scarcely defined Japanese target populations indicated significant mortality reductions, but these results are yet to be confirmed in western countries. To ascertain whether CXR screening decreases LC mortality at community level, we studied a clearly defined population-based cohort of smokers invited to screening. We present here the LC detection results and the 10-year survival rates.

**Methods:**

The cohort of all smokers of > 10 pack-years resident in 50 communities of Varese, screening-eligible (n = 5,815), in July 1997 was invited to nonrandomized CXR screening. Self-selected participants (21% of cohort) underwent screening in addition to usual care; nonparticipants received usual care. The cohort was followed-up until December 2010. Kaplan-Meier LC-specific survival was estimated in participants, in nonparticipants, in the whole cohort, and in an uninvited, unscreened population (control group).

**Results:**

Over the initial 9.5 years of study, 67 LCs were diagnosed in screening participants (51% were screen-detected) and 178 in nonparticipants. The rates of stage I LC, resectability and 5-year survival were nearly twice as high in participants (32% stage I; 48% resected; 30.5% 5-year survival) as in nonparticipants (17% stage I; 27% resected; 13.5% 5-year survival). There were no bronchioloalveolar carcinomas among screen-detected cancers, and median volume doubling time of incidence screen-detected LCs was 80 days (range, 44-318), suggesting that screening overdiagnosis was minimal. The 10-year LC-specific survival was greater in screening participants than in nonparticipants (log-rank, *p *= 0.005), and greater in the whole cohort invited to screening than in the control group (log-rank, *p *= 0.001). This favourable long-term effect was independently related to CXR screening exposure.

**Conclusion:**

In the setting of CXR screening offered to a population-based cohort of smokers, screening participants who were diagnosed with LC had more frequently early-stage resectable disease and significantly enhanced long-term LC survival. These results translated into enhanced 10-year LC survival, independently related to CXR screening exposure, in the entire population-based cohort. Whether increased long-term LC-specific survival in the cohort corresponds to mortality reduction remains to be evaluated.

**Trial registration number:**

ISRCTN90639073

## Background

The historical observational studies of chest x-ray (CXR) screening for lung cancer (LC) showed improved LC-specific survival but no reduction of LC-specific mortality [1-6]. Four case-control studies performed in the 1990s in Japan on a population level however suggested CXR screening effectiveness, as indicated by LC mortality reductions of about 40% [7,8]. The results of these case-control studies were obtained in scarcely defined target populations, were likely biased by self-selection and are yet to be confirmed in western countries. Randomized trials of LC screening by chest radiography performed in the 1970s [9,10] failed to answer the question of efficacy of radiographic screening, likely due to methodological flaws [11]; however, the possibility was recognized that a small but important benefit from annual CXR could have been missed [12-14]. The lung component of the Prostate, Lung, Colon and Ovarian (PLCO) cancer screening randomized trial was carried out to definitively assess the efficacy of CXR screening [15]. The recently published PLCO results showed that four annual chest radiographs did not reduce LC-specific mortality in volunteers [16], but it remains uncertain whether this finding may be generalized on a population level.

In 1997 we started to study the PREDICA cohort (hereafter concisely referred to as "cohort"), a clearly defined population-based cohort of 5,815 heavy or long-term smokers of the Province of Varese, Italy, to ascertain whether CXR screening at community level decreases LC mortality [17]. The cohort was invited to an annual CXR screening program and was followed-up for 13.5 years. Our aim is to evaluate here the LC detection results and the long-term LC survival in screening participants as well as in nonparticipants of this population-based cohort. We also examine LC survival by intent-to-screen, in the entire cohort invited to screening. This analysis is preliminary to the evaluation of CXR screening effectiveness on a population level by the LC mortality indicator, to be reported at a later date.

## Methods

### Summary of study design

The design is a nonrandomized LC screening study of a defined population-based cohort of asymptomatic smokers. In July 1997 the cohort was offered annual CXR screening for 4 years and was observed for 13.5 years. The individuals who self-selected to participate underwent screening in addition to the Italian National Health System (NHS) usual care; nonparticipants received usual care only. At the end of the active screening period, we evaluated the rate of positive CXR screens, the LC yield, and the invasive procedures performed to follow-up radiographic abnormalities. Moreover, the clinico-pathological features of all LCs diagnosed in the initial 9.5 years of study were examined in participants and in nonparticipants. The whole cohort was subsequently followed-up until study cut off, December 31, 2010. The main outcome was LC specific survival from time of cancer diagnosis in participants, in nonparticipants, and in the whole cohort relative to a control group. Secondary outcomes were the LC detection results.

### Cohort (n = 5,815)

Recruitment modalities and characteristics of the cohort have been described [17]. Briefly, recruitment was effected by 50 general practitioners (GP) physicians collaborating on the study, who served altogether a population of about 60,000 adults in communities widely spread over the Varese Province. In early 1997 the GPs compiled a preliminary recruitment list, encrypted to comply with privacy regulations, pooling all smokers resident in the communities of their practice who met the screening criteria: cigarette smoker of > 10 pack-years (current smoker or ex-smoker for < 10 years), resident in the Varese Province, both genders, aged 45-75 years, fit for possible thoracotomy, without symptoms (bloody or worsening cough, hoarseness, unexplained weight loss) and without diagnosed or suspected LC. No other exclusion criteria were used. The 50 GPs served nearly 100% of residents in their respective communities, in the NHS. They recruited all cigarette smokers in their public medical practices who were screening-eligible, based on self-reported smoking history abstracted from medical records. Completeness of the recruitment list was ascertained by the quality assurance team of the project (AI, MP, VD, and APa). Finally, the cohort consisted of 5,815 screening-eligible smokers. Their mean age was 56.6 years (8.3 SD), males were 73.7%, current smokers were 76.3% and the median pack-years smoked was 32.8 (interquartile range (IQR), 22.8-46.0) [17]. Targeted subjects were told nothing of the study until the screening recruitment list was complete. On May 1997 a letter informing all cohort subjects about risks and possible benefits of CXR screening (significantly increased resectability and survivorship of screen-detected lung cancer) was sent out. The letter also served as invitation to participate in LC screening free of charge. Moreover, the screening project was widely publicized in the local media. Signed informed consent was obtained from all individuals who accepted to participate in screening. For nonparticipants, informed consent was waived and their names were encrypted, as approved by the Varese Hospital and Health District Ethics Committee.

### Screening participants and timeline of study

The study lasted from July 1, 1997 until December 31, 2010. Enrolment of screening participants extended for 4.5 years, until December 31, 2001, although it concentrated early in the study (median enrolment, February 1998). As published in the initial report of the cohort, 1,244 subjects (21% of the cohort) undertook at least the baseline CXR screening by December 31, 2001 and were allocated as participants [17]. The other 4,571 subjects were nonparticipants. All cohort individuals were observed from start of study until December 31, 2010 when the study was cut off, or until date of death, whichever came first. We examined the LC detection results and the survival of all LCs diagnosed in the cohort during the period from July 1, 1997 until December 31, 2006, giving each survivor a potential minimum follow-up of 48 months.

### CXR screening and management of positive screens

The screening protocol consisted of baseline CXR and annual repeat screen for 4 years. Chest radiographs were taken as described previously [17] and were interpreted with single reading by one of three senior thoracic radiologists, in comparison with previous images if available. A CXR was defined positive when a nodule, mass or infiltrate was identified as suspicious for LC, at the radiologist's discretion. After undertaking the baseline screen, participants with negative CXR reading were scheduled for subsequent annual appointments and received an annual reminder. Participants with positive CXR screens, and all subjects in the cohort with nonscreen-detected abnormalities suspicious for LC, were referred to the thoracic oncologists of the Varese University Hospital for diagnostic follow-up. The standard of care in Varese for the investigation of suspicious abnormalities was used, initially consisting of repeat imaging by CXR or computed tomography (CT) after 1-month to 6-month interval, depending on the degree of LC suspect. If necessary, histologic/cytologic diagnosis was obtained by the least invasive method, chosen among fine-needle aspiration cytology (FNAC), bronchoscopic biopsy and video-assisted thoracoscopic (VATS) lung wedge resection. Regardless of the detection modality, by screening or outside screening, the LCs diagnosed in this study were treated by usual international criteria [18]. Management of LCs was centralized in the Varese University Hospital, with the exception of very few patients treated at nearby private hospitals essentially with the same standard of care. All individuals with suspect LC who were candidate for active cancer treatment had histological/cytological confirmation of diagnosis and the LCs were staged according to the 6^th ^ed. of TNM Classification of Malignant Tumors [19]. The LC diagnosis was clinico-radiologic in patients who refused biopsy or treatment and in those subjects who were candidates for supportive care only. Except for patients scheduled for induction chemo- or radiotherapy, LC cases candidate to surgery were operated within one month from diagnosis. We prospectively recorded all LCs diagnosed in the cohort during the initial 9.5 years of study, adopting the Varese Cancer Registry criteria [17,20], by linkage with the Epidemiology Observatory [21], Varese Cancer Registry, Varese Province hospital records and pathology records. In screening participants the LCs were classified either as screen-detected or nonscreen-detected (i.e. diagnosed as interval cases or outside of screening). We recorded the size of screen-detected cancers and calculated the volume doubling time (VDT) of cancers diagnosed at incidence screening, based on tumor size measurements from sequential CXRs. To calculate VDT of a lung tumor not evident retrospectively on the prior radiograph, the tumor was arbitrarily attributed the dimension of 6 mm on the prior CXR, corresponding to the estimated visibility threshold [22]. Follow-up included review of medical records for collection of data concerning LC histology, stage at diagnosis and treatment. The LC deaths occurred in the cohort during the 13.5 year observation period were searched by linkage with the Varese Mortality Registry, last accessed on April 27, 2011. Date and cause of death were obtained from death certificates of the Registry. For subjects who had migrated within the Lombardy region, survival status were ascertained by linkage with the Lombardy Health Registry of residents [23]; for subjects not traceable through this registry, investigations were done among persons' next-of-kin and via demographic services. Death certificates were reviewed by the mortality review committee members (LD, AI, NR, FS, SP, APo and WM), who were not blinded to the mode of LC detection. Those deaths definitely attributed to LC we recorded as LC-specific deaths.

### Control LC group

A control group of uninvited and unscreened LC patients prospectively followed-up was identified, to be used as comparator for LC cases found in the cohort. To that effect we accessed the database of all LC cases (n = 243) diagnosed during the calendar year 2000 in the 350,000 residents of the Varese district area. The year 2000 LC cases were chosen because the demographic and clinico-pathological data of all these patients were available and published [18,24], and their 10-year follow-up was obtained by linkage with Varese Epidemiology Observatory [21]. Of the 243 LC patients diagnosed in 2000, 156 subjects met the screening criteria as of July 1997 (birth-year cohort, smoking history, residence in the district of the cohort, uninvited to screening and unscreened) and constituted the control LC group of the present study. The cause of death of the control LC group subjects was assigned by the mortality review committee, as described above for deceased subjects of the cohort.

### Statistical analysis

Continuous variables were reported as mean with standard deviation (SD), or median with range or interquartile range (IQR, 25th-75th percentiles). Categorical data were presented as numbers and percentages. The comparison between groups was made using Student's *t*-test, or Mann-Whitney *U *test, for continuous variables. Categorical data were compared by Chi-square test, or by Fischer exact test if necessary. LC survival was defined as the time from LC diagnosis to LC-related death and was censored at the last follow-up date (December 31, 2010) if no events occurred. In the survival analysis, follow-up time was stopped at 120 months. Survival probability was estimated according to the Kaplan-Meier method, and log-rank test was used for comparison of survival between groups. Multivariate analysis was performed by the Cox regression model to evaluate the relative role of different screening exposures in predicting prognosis. Gender, age, pack-years and LC histology were considered as potential confounders. All tests were two-sided. *P *values < 0.05 were considered statistically significant. Analysis was performed at the Department of Public Health and Community Medicine, University of Verona (APo, WM), using Stata software 11.0 (Stata Corporation, College Station, Texas, USA).

This study was approved by the Varese Hospital and Health District Ethics Committee.

## Results

After baseline screening, the absolute number and the proportion of participants in each annual round (T) were: T1, 1,046 (84.1%); T2, 876 (70.4%); T3, 735 (59.1%); T4, 566 (45.5%). In addition, we traced 429 subjects (34.5% of participants) who completed the planned screening rounds and after discussion with their physician continued annual CXR screening. There were 1,114 of these additional annual CXR screens performed until December 31, 2006, and their outcomes were included in the analysis of results. Some of the participants cooperated incompletely, slightly extending the interval between screens, or not attending scheduled exams. Overall, 5,581 screening tests were performed (baseline, n = 1,244; incidence, n = 4,337). The median number of CXR screenings done by each participant, including the baseline and the above mentioned additional tests, was 4 (IQR, 2-7).

### Evaluation of positive screens (Table 1)

**Table 1:** Positive screens, invasive follow-up procedures and screen-detected lung cancers

	Baseline Screening (n = 1,244)	Annual Incidence Screening (n = 4,337)	All Screens (n = 5,581)
**Positive screens, n (%)**	54 (4.34)	170 (3.92)	224 (4.01)

**Any invasive procedure, n (%)**	15 (1.21)	27 (0.62)	42 (0.75)

**Invasive procedure for benign lesion, n (%)**	3 (0.24)	6 (0.14)	9 (0.16)

**Screen-detected cancers, n (%)**	13* (1.04)	21 (0.48)	34* (0.61)

*1 patient: positive sputum cytology

Of the total 5,581 CXR exams performed, 224 (4.01%) were interpreted as positive. The rate of positive tests was 4.34% (54 of 1,244 participants) at baseline and 3.92% (170 of 4,337) at annual screens. Of the total 224 positive screens, 190 (84.8%) were false-positive (3.4% of the 5,581 tests performed in total). One FNAC, 8 VATS lung wedge resections and no lobectomies were done to biopsy 8 lesions that were benign (3 hamartomas, 3 fibroses, 1 infarct, 1 pneumonia). Overall the rate of invasive diagnoses for benign lesions was 1.6/1,000 CXR exams.

There were 34 true positive CXRs (0.61%); in 24 cases (72%) the LCs were peripherally located. The LC detection rate was 1.04% (13 of 1,244) at baseline screening, and 0.48% (21 of 4,337) at annual screens.

### Lung cancers detected in the cohort and in the control group

Overall, 245 LC patients were diagnosed in the cohort. Table 2 shows their demographics, pack-years, proportion of asymptomatic diagnoses, LC histology and stage distribution, compared with the corresponding data of control group LC patients. Characteristics of LC patients were similar in the cohort and in the control group, except for a slight preponderance of adenocarcinomas in the cohort (33% vs. 27%). The proportion of asymptomatic diagnoses was higher in the cohort (27.3% vs. 18.6%), as expected. The characteristics of participants and nonparticipants who were diagnosed with LC are shown in Table 3 and Table 4. There were 67 LCs found in the 1,244 screening participants and 178 LCs in the 4,571 nonparticipants. Of the LCs diagnosed in participants, 34 (51%) were screen-detected and 33 (49%) were nonscreen-detected (interval cases and cases diagnosed outside screening). In the 34 screen-detected LCs, mean tumor size was 3.1 ± 1.6 cm; in the 18 screen-detected LCs in stage I, the mean size was 2.9 ± 1.1 cm. The VDT was calculated for all 21 non-baseline screen-detected cases. For 16 of these cases the lung tumors were not evident retrospectively on the prior radiograph and were arbitrarily attributed the 6 mm estimated visibility threshold dimension. The median VDT of all non-baseline screen detected cases was 80 days (range: 44-318); only one of these cancers had VDT > 300 days.

**Table 2:** Characteristics of lung cancer (LC) patients in the cohort and in the control group

	Patients with LC		
	**Cohort** **(n = 245)**	**Control Group** **(n = 156)**	***P***

**Age, median (IQR)**	68 (60-73)	68 (62-73)	0.930

**Male/female (ratio)**	221/24 (9.2)	144/12 (12.0)	0.472

**Pack-years, median (IQR)**	49 (36-66)^a^	45 (30-67)^b^	0.598

Asymptomatic LC diagnosis*, n (%)	67 (27.3%)	27 (18.6%)^c^	0.051

**Histologically confirmed LC, n (%)**	211 (86%)	131(84%)	

*Adenocarcinoma, n (%)*	69 (33%)	36 (27%)	

*Squamous cell carcinoma, n (%)*	82 (39%)	64 (49%)	0.411

*Other NSCLC, n (%)*	31 (15%)	14 (11%)	

*SCLC, n (%)*	29 (14%)	17 (13%)	

**Stage of LC at diagnosis^d, e^**			

*I, n (%)*	49 (21%)	23 (15%)	

*II, n (%)*	13 (6%)	9 (6%)	

*IIIA, n (%)*	30 (13%)	20 (13%)	0.433

*IIIB-IV and extensive SCLC, n (%)*	136 (60%)	102 (66%)	

Follow-up months, median (range)^f^	101 (49-161)	123 (119-131)	0.232

*LC*: lung cancer; *IQR*: interquartile range; *NSCLC*: non-small cell lung cancer; *SCLC*: small cell lung cancer

*In nonparticipants of the cohort and in the control group the asymptomatic LCs diagnosed were all incidentally found, as documented by patient file review

^a^Not available in 28 patients

^b^Not available in 41 patients

^c^Not available in 11 patients

^d^Not available in 17 patients of Cohort

^e^Not available in 2 patients of Control Group

^f^Calculated in survivors at end of study

**Table 3:** Characteristics of screening participants and of nonparticipants who were diagnosed with lung cancer (LC)

	Participants with LC (n = 67)	Nonparticipants with LC (n = 178)	*P*
**Age, median (IQR)**	66 (60-72)	69 (61-74)	0.196

**Male/female (ratio)**	60/7 (8.6)	161/17 (9.5)	0.834

**Pack-years, median (IQR)**	48 (36-68)^a^	49 (36-66)^b^	0.768

Histologically confirmed LC, n (%)	63 (94%)	148 (83%)	0.047

*Adenocarcinoma, n (%)*	18 (29%)	51 (34%)	

*Squamous cell carcinoma, n (%)*	27 (43%)	54 (37%)	**0.573**

*Other NSCLC, n (%)*	7 (11%)	25 (17%)	

*SCLC, n (%)*	11 (17%)	18 (12%)	

**Stage of LC at diagnosis^c, d^**			

*I, n (%)*	21 (32%)	28 (17%)	

*II, n (%)*	3 (5%)	10 (6%)	

*IIIA, n (%)*	9 (14%)	21 (13%)	0.078

*IIIB-IV and extensive SCLC, n (%)*	32 (49%)	104 (64%)	

**Follow-up months, median (range)**^**e**^	95 (49-161)	104 (55-151)	**0.962**

*LC*: lung cancer; *IQR*: interquartile range; *NSCLC: *non-small cell lung cancer; *SCLC*: small cell lung cancer

^a^Not available: 5 patients

^b^Not available: 23 patients

^c^Not available in 2 Participants

^d^Not available in 15 Nonparticipants

^e^Calculated in survivors at end of study

**Table 4:** Characteristics of participants with screen-detected lung cancer (LC) and with nonscreen-detected LC

	Participants with LC		
	**Screen-detected** **(n = 34)**	**Nonscreen-detected** **(n = 33)**	***P***

**Age, median (IQR)**	66 (60-71)	69 (59-72)	0.965

**Male/female (ratio)**	30/4 (7.5)	30/3 (10)	0.721

**Pack-years, median (IQR)**	55 (39-77)^a^	46 (33-76)^b^	0.200

Histologically confirmed LC, n (%)	34 (100%)	29 (88%)	0.036

*Adenocarcinoma, n (%)*	11 (32%)	6 (21%)	

*Squamous cell carcinoma, n (%)*	16 (47%)	12 (41%)	0.618

*Other NSCLC, n (%)*	3 (9%)	4 (14%)	

*SCLC, n (%)*	4 (12%)	7 (24%)	

**Stage of LC at diagnosis^c^**			

*I, n (%)*	18 (53%)	3 (9%)	

*II, n (%)*	2 (6%)	1 (3%)	

*IIIA, n (%)*	5 (15%)	4 (12%)	< 0.0001

*IIIB-IV and extensive SCLC, n (%)*	7 (21%)	25 (76%)	

Indeterminate, n (%)*	2 (6%)	0	

**Follow-up months, median (range)**^**d**^	100 (49-161)	72 and 98^e^	**-**

*LC*: lung cancer; *IQR*: interquartile range; *NSCLC*: non-small cell lung cancer; *SCLC*: small cell lung cancer

^a^Not available: 2 patients

^b^Not available: 3 patients

^c^Not available in 1 Nonscreen-detected Participant

*Patient refused to ascertain stage

^d^Calculated in survivors at end of study

^e^Only two survivors

Patients with screen-detected and nonscreen-detected LC were similar for age, gender distribution and pack-years, as shown in Table 4. Of the screen-detected cancers, 32% were adenocarcinomas, none was bronchioloalveolar carcinoma; 47% were squamous cell carcinomas. Among nonscreen-detected cancers the proportion of small cell LCs was twice as high as in screen-detected (24% vs. 12%), and the LC stage distribution was significantly different from that of screen-detected cancers *(P *< 0.0001). Table 5 shows the stage distribution of screen-detected LCs. Stage I cancers were 53% (46% at baseline; 57% at annual tests). The LC resection rate was significantly higher in screen-detected relative to nonscreen-detected cases (76% vs. 18%; *P *< 0.0001), and in participants relative to nonparticipants (48% vs. 27%; *P *= 0.002). Interestingly, the LC resection rate was essentially identical in nonparticipants and in the control group (27% vs. 26%; *P *= 0.887). Among the 26 resected screen-detected LC patients there were no hospital deaths. Follow-up of LC cases was 100% complete both in the cohort and in the control group; median follow-up was 101 months (range, 49-161) and 123 months (119-131), respectively (Table 2).

**Table 5:** Stage distribution of screen-detected lung cancers (LCs)

LC Stage at Diagnosis	Baseline Screening (LCs, n = 13)	Annual Screening After Baseline (LCs, n = 21)	All Screens (LCs, n = 34)
**I, n (%)**	6 (46)	12 (57)	18 (53)

**II, n (%)**	1 (8)	1 (5)	2 (6)

**IIIA, n (%)**	2 (15)	3 (14)	5 (15)

**IIIB-IV, n (%)**	3 (23)	4 (19)	7 (21)

Indeterminate, n (%)*	1 (8)	1 (5)	2 (6)

*LC*: lung cancer

*2 patients refused to complete staging procedures

### LC specific survival

**Figure 1:**
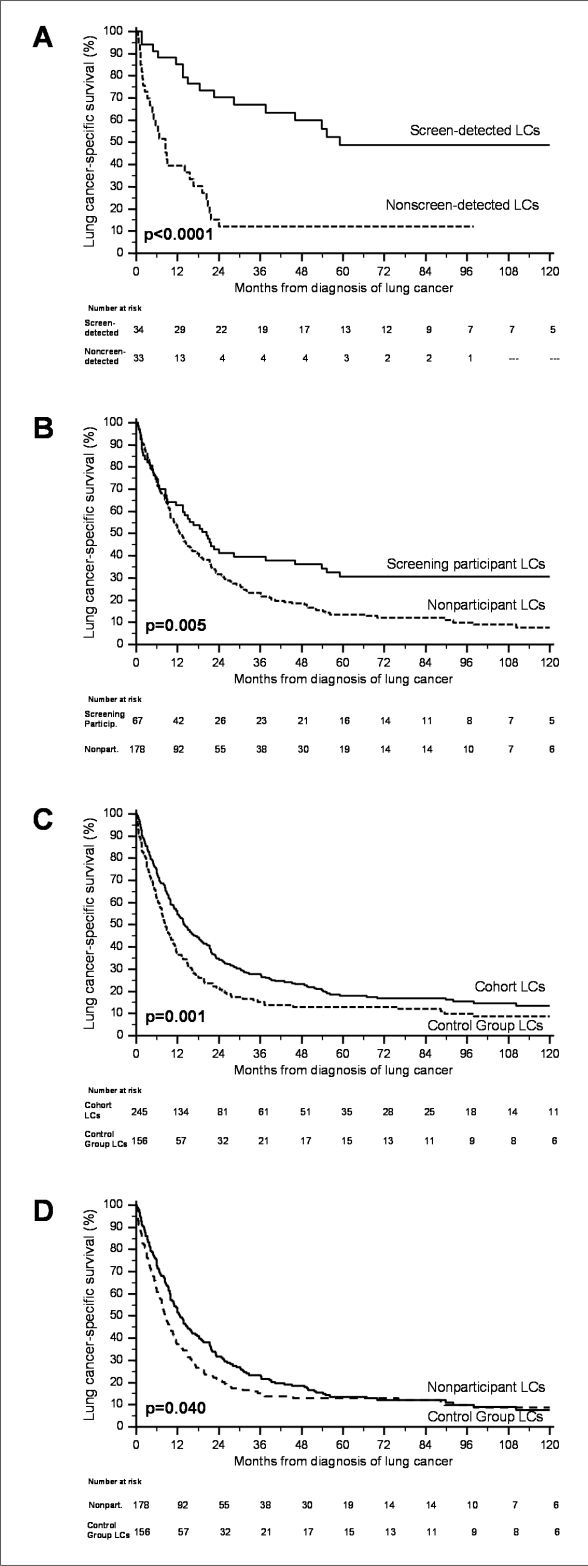
**Comparison of lung cancer (LC) -specific survival curves (Kaplan-Meier)**. Shown are **(A) **screen-detected LCs (n = 34) vs. nonscreen-detected LCs (n = 33), **(B) **screening participant LCs (n = 67) vs. nonparticipant LCs (n = 178), **(C) **cohort LCs (n = 245) vs. control group LCs (n = 156), and **(D) **nonparticipant LCs (n = 178) vs. control group LCs (n = 156).

**Figure 2:**
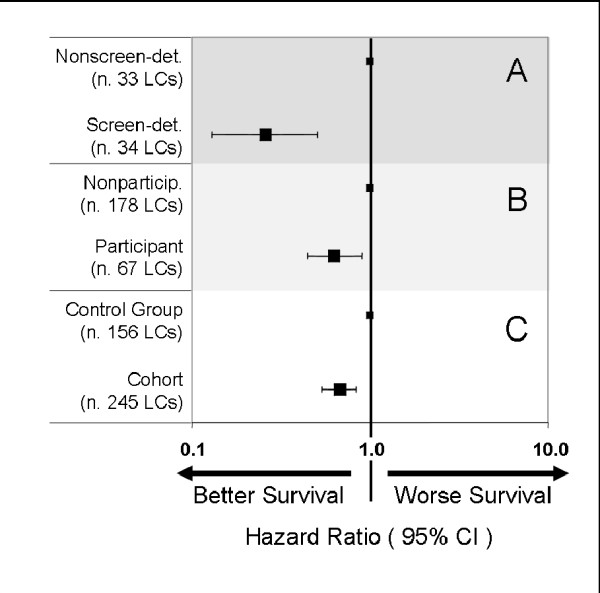
**Multivariate analysis (Cox-proportional hazard model) of the impact of lung cancer (LC) screening on survival**. Shown are **(A) **analysis of 67 LCs of participants (screen- vs. nonscreen-detected), **(B) **analysis of 245 LCs of the cohort (participants vs. nonparticipants), and **(C) **analysis of 401 LCs (156 LCs of control group vs. 245 LCs of the cohort). All the models were adjusted for gender, age, pack-years and histology.

Kaplan-Meier survival analysis showed significantly greater LC survival in screen-detected cases than in nonscreen-detected (log-rank, *P *< 0.0001; Figure 1A), in participants than in nonparticipants (log-rank, *P *= 0.005; Figure 1B), and in the whole cohort than in the control group (log-rank, *P *= 0.001; Figure 1C). Median LC specific survival was 14.1 months (95% confidence interval (CI), 10.9-17.2) in the whole cohort and 8.4 months (95%CI, 6.6-10.2; *P *= 0.001) in the controls. The LC survival curve of the cohort and of the control group plateaued after five years, showing persistently higher survival in the cohort than in the control group, until 10-year follow-up (Figure 1C). Moreover, comparing controls versus nonparticipants (neither group was screened) we found that after 5 years from diagnosis the LC survival in these two groups was essentially identical (Figure 1D and Table 6). All 10-year survivors had undergone cancer resection. The 2-year, 5-year and 10-year LC-specific survivals in all groups are shown in Table 6. In unadjusted Cox regression, the risk of death was lower in screen-detected LC patients relative to nonscreen-detected (*P *< 0.0001), in participants relative to nonparticipants (*P *= 0.013), in the whole cohort relative to the control group (hazard ratio (HR) = 0.69; 95%CI, 0.55-0.87; *P *= 0.002) (Table 7). After adjusting for gender, age, pack-years and histology, with Cox multivariate analysis the risk of death in the whole cohort remained substantially unchanged (HR = 0.70; 95%CI, 0.56-0.87; *P *= 0.001) (Figure 2). In the cohort, 11 subjects were 10-year survivors: 6 of 178 nonparticipants (3.4%), 5 of 34 screen-detected participants (14.7%) and none of nonscreen-detected participants. The 10-year survivors in the control group were 4.5%.

**Table 6:** Lung cancer (LC)-specific Kaplan-Meier survival rates at 2, 5 and 10 years from LC diagnosis

	LC Specific Survival		
**LC Patient Group**	**2-year Survival (%)**	**5-year Survival (%)**	**10-year Survival (%)**

**Screen-detected (n = 34)**	70.2	48.6	48.6

**Nonscreen-detected (n = 33)**	12.1	12.1	---

**Participants (n = 67)**	41.2	30.5	30.5

**Nonparticipants (n = 178)**	31.6	13.5	7.6

**Cohort (n = 245)**	34.3	18.0	13.3

**Control Group (n = 156)**	21.5	13.0	8.7

*LC*: lung cancer

**Table 7:** Univariate survival analysis of clinico-pathological factors (unadjusted Cox proportional hazard model)

		A		B		C
		**n = 67**		**n = 245**		**n = 401**

		**HR**		**HR**		**HR**

		**(95% CI)**		**(95% CI)**		**(95% CI)**

**Gender**						

Female		1.00		1.00		1.00

		(reference)		(reference)		(reference)

Male		1.42		0.85		0.91

		(0.51-3.97)		(0.54-1.34)		(0.63-1.32)

**Age at diagn. (+1 y)**		1.01		1.01		1.01

		(0.97-1.06)		(0.99-1.03)		(0.99-1.03)

**Pack/years (+1)**		1.00		1.00		1.00

		(0.99-1.01)		(0.99-1.01)		(1.00-1.01)

**Histology**						

Adenocarcinoma		1.00		1.00		1.00

		(reference)		(reference)		(reference)

Squamous cell ca		1.37		0.97		1.19

		(0.61-3.08)		(0.67-1.39)		(0.9-1.58)

Other NSCLC		2.21		1.85 *		1.90 *

		(0.74-6.62)		(1.15-2.97)		(1.29-2.79)

SCLC		2.45 *		1.79 *		1.69 *

		(1.00-6.1)		(1.12-2.86)		(1.17-2.46)

CDO		5.60 *		2.22 *		2.44 *

		(1.7-18.46)		(1.44-3.45)		(1.73-3.42)

**Stage**						

I		1.00		1.00		1.00

		(reference)		(reference)		(reference)

II		1.56		2.32 *		3.31 *

		(0.19-12.98)		(1.07-5.05)		(1.85-5.92)

IIIA		5.94 *		4.22 *		4.08 *

		(1.93-18.26)		(2.37-7.53)		(2.56-6.51)

IIIB - IV		14.02 *		8.38 *		8.35 *

		(5.58-35.24)		(5.2-13.51)		(5.65-12.33)

Indeterminate		5.21		2.54 *		2.79 *

		(0.61-44.11)		(1.29-5.02)		(1.51-5.13)

Unexposed	**33 LCs**	1.00	**178 LCs**	1.00	**156 LCs**	1.00

		(reference)		(reference)		(reference)

Exposed	**34 LCs**	0.26 **	**67 LCs**	0.62 ***	**245 LCs**	0.69 ****

		(0.14-0.49)		(0.44-0.86)		(0.55-0.87)

Shown are **(A) **67 lung cancers (LCs) of screening participants, **(B) **245 LCs of the cohort, and **(C) **401 LCs (156 of control group and 245 of cohort)

*HR*: hazard ratio; *CI*: confidence interval; *NSCLC*: non-small cell lung cancer; *SCLC*: small cell lung cancer; *CDO*: clinical diagnosis only; *LC*: lung cancer

*P < 0.0001

**P < 0.0001 (34 LCs Screen-detected vs. 33 LCs Nonscreen-detected)

***P = 0.0013 (67 LCs of Participants vs. 178 LCs of Nonparticipants)

****P = 0.002 (245 LCs of Cohort vs. 156 LCs of Control Group)

## Discussion

The impact of LC screening on a population level has been scarcely investigated. The generally low participation rate in LC screenings (11-35%) [17,25,26] and the volunteer effect bias [17,27] raise concern about the generalizability to the population of interest at community level of the results of randomized LC screening trials of highly selected volunteers. As an example, the recently completed National Lung Screening Trial, (NLST) showed 20.3% mortality reduction after CT screening in selected volunteers; however only about 7% of smokers in the United States would meet the NLST criteria [28]. Here we analyze the LC detection results and the LC survival rates at community level, in the setting of CXR screening offered to a clearly defined population-based cohort of smokers. Only 21% of this cohort participated in screening, a low attendance rate similar to that observed in mass screening for LC in Japan [29,30]. Analyzing the demographic features and risk factors of the whole screening-invited cohort, we previously reported that participation was possibly prompted by increased awareness of LC risk, while it was not related to educational level [17].

Overall, the LC yield of our CXR screening (0.61%) was similar to that recorded in the PLCO study (0.7%) [31] and in the Lung Screening Study (0.68%) [32]; the slightly lower LC yield in our screening was likely due to lower pack-years and lower compliance. In participants, we found that only about half (51%) of LCs were screen-detected, a proportion similar to that recorded in the PLCO trial (53%) [31]. In the other half of participants, LCs were symptom-detected and survival was very poor. Overall in screening participants the rates of stage I LC diagnosis, LC resection and 5-year survival were nearly twice as high as in nonparticipants, confirming the significantly increased resectability and survival achievable with CXR screening [7]. Notably, the Kaplan-Meier 10-year LC survival in the whole cohort was significantly greater than in the control group, and by multivariate Cox analysis this survival difference was independently related to CXR screening exposure, also after adjusting for age, gender, pack-years and histology. Improved LC survival persisting over 10 years in the population-based cohort of this study suggests mortality reduction, however no conclusion can be drawn about the effectiveness of CXR screening, because survival may be biased by lead-time, selection, length-time and overdiagnosis.

We addressed the potential impact of these biases. In the context of our study the impact of lead-time bias on long-term LC specific survival of participants seems negligible, because the LC specific survival curve of screening participants plateaued after 5 years (Figure 1B). Enrolment of asymptomatic individuals in the cohort was a source of healthy selection. The latter however did not influence the long-term LC survival, as shown by comparison of the Kaplan-Meier LC survival curve of cohort's nonparticipants and of control group (Figure 1D); the LC survival was initially greater in nonparticipants, likely due to healthy selection, but after 5 years from diagnosis it was virtually identical to that of control group (Figure 1D and Table 6). The latter finding is also consistent with the similar LC resection rate (27% vs. 26%) and similar proportion of histological diagnoses and of LC stage distribution in the nonparticipants and in the control group (Table 2 and Table 3). To evaluate length-time bias and overdiagnosis bias in our study, we focused on the VDT of screen-detected LCs, that is also an indicator of tumor aggressiveness [33-35]. Incidence screen-detected LCs had short median VDT (80 days), and only one of these cancers had VDT > 300 days, indicating that most of them grew rapidly and unlikely were overdiagnosed, in agreement with the observations of other authors about CXR screen-detected LCs [22]. Moreover, among screen-detected cancers we found no cases of bronchioloalveolar carcinoma, a slow-growing subtype that may be overdiagnosed. Furthermore, we previously showed that the LC incidence standardized rate ratio in the whole cohort was 1.07 [17], suggesting that the number of possibly overdiagnosed LCs was minimal.

This study has limitations. The long duration of participants' enrolment may slightly underestimate the nonparticipants' survival [17]. Compliance with annual screening progressively decreased, more markedly than in other CXR screening trials. By year 3 we recorded 59% adherence, while 79% was observed in the PLCO radiography screening [16] and about 80% in the Mayo Lung Project [36]. In our study the median number of CXR screenings done by each participant was four, instead of five expected. Sub-optimal compliance along with low participation rate possibly compromised the effectiveness of screening, an issue that will be addressed in a separate paper. Another limitation is that the investigators assigning the cause of death in the cohort and in the control LC group were not blinded to mode of LC detection. Sensitivity of LC death certificates however was shown to be high and similar in screening participants and nonparticipants [17]; therefore, selective misclassification of the cause of death unlikely occurred. A relevant question is whether the 156 patients of the control LC group are an appropriate control for the LC patients found in the cohort. The control LC group source were all smokers resident in the Varese district who were diagnosed with LC during the calendar year 2000 and who met the screening criteria as of July 1997; therefore comparison with the cohort, enrolled in 1997 and representing well the Varese smokers population [17] appears meaningful. We ruled out the possibility of period-effect for the year 2000 LC survival, because differences of LC survival in the cohort by pair-wise years of cancer diagnosis (1997-1998, 1999-2000, 2001-2002, 2003-2004, 2005-2006) were not significant (log-rank, *P *= 0.401). Indeed the control group LCs closely represented the cohort LCs as for age, gender, pack-years, proportion of histologically confirmed LCs, distribution of LC types and stages, and duration of follow-up (Table 2). Moreover, for the evaluation of long-term survival of the cohort, the control group seems appropriate because the cohort's nonparticipants and the controls (neither group was screened) had similar long-term survival (Table 6). Another limitation is the small number of screen-detected LC cases; their survival rates therefore must be interpreted with caution. Strong features of this study are the completeness of the list of all eligible smokers resident in the 50 widely scattered communities invited to screening, and the completeness of follow-up of all LCs of the cohort and of the control group.

## Conclusion

This report documents increased 10-year LC-specific survival in an entire population-based cohort of 5,815 smokers invited to CXR screening at community level. This favourable long-term effect was obtained in spite of low (21%) screening participation rate and was independently related to CXR screening exposure. Whether enhanced LC-specific survival in the cohort corresponds to mortality reduction remains to be evaluated.

## Competing interests

The authors declare that they have no competing interests.

## Authors' contributions

LD, AI and APo were responsible for study development and wrote the report. AI, MP, VD, VC and APa carried out entry and protection of data and quality assurance monitoring. The mortality review committee responsible for re-evaluation of death certificates and final classification of causes of death included clinicians (LD, AI, NR), a pathologist (FS), an epidemiologist (APo) and a statistician (WM). All authors read and approved the final manuscript.

## Pre-publication history

The pre-publication history for this paper can be accessed here:

http://www.biomedcentral.com/1471-2407/12/18/prepub
